# Computed tomographic evaluation of pectus excavatum in 14 cats

**DOI:** 10.1371/journal.pone.0262866

**Published:** 2022-01-21

**Authors:** Renata Komsta, Anna Łojszczyk, Piotr Dębiak, Piotr Twardowski, Barbara Lisiak

**Affiliations:** Laboratory for Radiology and Ultrasonography, Department and Clinic of Animal Surgery, Faculty of Veterinary Medicine, University of Life Science, Lublin, Poland; University of Liverpool, UNITED KINGDOM

## Abstract

Pectus excavatum (PE) is one of the most frequently reported chest deformities. However, limited studies are available with regard to its CT scan findings in cats. In the present research computed tomographic images of the thoraxes of 14 cats diagnosed with PE has been reviewed. This is one of the first studies exploring the use of CT to characterise PE in animals. The aim of this study was to present characteristic CT features of PE in cats. The introduction of new criteria for better assessing thoracic wall deformity–a correction index (CI) and an asymmetry index (AI)–was also proposed. The study revealed a high variety of morphological features of PE in cats. It was demonstrated that among the 14 cats: cranial PE (an atypical location) occurred in seven cats while seven cats had typical (caudal) PE, long PE occurred in five cats, while short PE had nine cats. Of the 14 cats included in the study eight showed symmetric PE, and asymmetric PE was found insix. Thoracic asymmetry was found in six cats. Six cats had sternal torsion. Based on the Vertebral Index moderate or severe PE was revealed in 11 animals. In the group of cats studied the CI ranged from 12.20 to 32.11. The magnitude of AI did not exceed 10% in any of the cats studied. The study confirmed statistically significant differences in the CI values between groups of cats with different degrees of PE severity (p = 0.02). CT examination showed many PE features that have not been discussed so far. The main benefit of CT examination is its ability to reveal asymmetric PE, thoracic asymmetry and sternal torsion. CI and AI provided a clinically useful tool to quantify thoracic wall deformity in order to obtain comparable results between cats with PE.

## Introduction

Pectus excavatum (PE) is a congenital anomaly of the thoracic wall, characterised by the inward deviation of the sternum and costal cartilage with a subsequent decrease in thoracic dorsoventral diameter [1–4]. PE is the most commonly described thoracic deformity in humans and animals. In severe forms, PE is associated with cardiac disorders and respiratory failure [2, 4–9]. Many descriptions of PE in cats can be found in veterinary literature; however, most of them refer to diagnosis and surgical correction of PE depending on clinical and radiographic diagnosis [6–17]. Radiographic classification of the degree of PE in cats and dogs is determined with the use of the frontosagittal index (FSI) and the vertebral index (VI) [5, 10, 14]. The FSI is the ratio between the width of the thorax at the 10^th^ thoracic vertebra and the distance from the centre of the ventral surface of the 10^th^ vertebral body to the nearest point on the sternum. The VI is the ratio of the distance between the centre of the dorsal surface of the vertebral body overlying the deformity to the nearest point on the sternum and the dorsoventral diameter of the same vertebral body [5, 10, 14]. These indices are used in human practice and adopted for use in veterinary medicine [11, 18]. However, studies have shown no correlation between the clinical severity and degree of PE calculated on the basis of FSI and VI [6, 19–23]. In humans, it has been shown that the FSI largely depends on the shape of the chest. In individuals with a barrel-shaped chest, the measurement dependent on chest width may be misleading [19–22]. In dogs diagnosed with PE, it has been documented that the VI is a more reliable gauge of the degree of thoracic depth reduction compared to the FSI [23]. In cats and brachycephalic dogs it has been demonstrated that the clinical severity of PE is dependent on the anatomic location of sternal deviation [6, 23]. PE may also contribute to thoracic asymmetry, which is difficult to accurately demonstrate through a radiological examination. Therefore, in recent years, there have been proposals to use thoracic CT to plan surgical reduction of PE [6, 21, 24, 25]. A detailed analysis of the characteristic PE features visible in CT scans in cats is lacking in veterinary literature.

The purpose of this retrospective study was to report CT findings in cats diagnosed with PE and evaluate two potential criteria that can be used to more precisely assesses thoracic deformity: a correction index (CI) and an asymmetry index (AI).

## Material and methods

### Ethics statement

In accordance with the Act “On the Protection of Animals Used for Scientific or Educational Purposes” (15/01/2015) this study was considered as sub-threshold for specific ethical approval, as the work involved only the analysis of data routinely recorded from normal and necessary clinical procedures.

### Cases

The digital medical records of cats that underwent CT examination (Philips MAX-16 slice unit, Philips Healthcare, Suzhou, PRC) in the Veterinary Clinic at the University of Life Sciences in Lublin were retrospectively reviewed from January 2016 to March 2020.

Inclusion criteria of CT scans used in the study included CT scans of cats with PE based on VI, including both sagittal and transverse CT scans covering the entire thorax, obtained while the cat positioned in sternal recumbency with the forelimbs pulled cranially. Cats diagnosed with thoracic spine deformities, thoracic wall trauma and diaphragmatic hernias were excluded. Decisions for the inclusion or exclusion of cats were made by a veterinary radiologist (RK). For each cat that met the inclusion criteria, the breed, age, and sex were recorded.

### Computed tomographic examination

The retrospective analysis of CT images was independently reviewed and analysed by three authors (RK, AŁ, PD) using multiplanar reconstruction images and electronic callipers integral to a DICOM PACS Acquisition Software workstation (Philips IntelliSpace Portal, Philips Medical Systems Nederland B.V., Bests, The Netherlands). All computed tomographic images were anonymised. The radiologists were blinded to clinical data of the cats.

All animals were sedated for CT examination with an injection of propofol (Scanofol 10 mg/ml, ScanVet, Gniezno, Poland, boluses until the effect). The cats were scanned without control of the respiratory phase.

The images were acquired with sharp and soft algorithms, in a bone enhanced window (window level 500 HU, window width 1600 HU). The image acquisition parameters were 120 kV, 120 mAs/slice, pitch 1.0069, slice thickness 1.0 mm, slice increment 0.5 mm, collimation 16*0.75 and rotation time 0.75 s.

**Fig 1:**
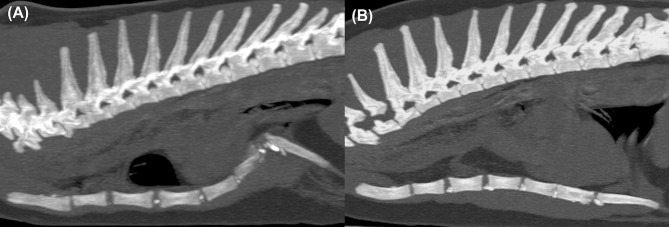
Sagittal multiplanar reconstruction CT images of the chests of two different cats. Image (A) shows typical (caudal) PE with short thoracic depression. Image (B) shows atypical (cranial) PE with long thoracic depression (the deepest point of ventrodorsal deviation of the sternum is located at the 3^rd^/4^th^ sternebrae).

Based on sagittal CT images of the cats’ thorax, the location and length of thoracic depressions were determined. The PE type was determined from the anatomic location of the deepest point of ventrodorsal deviation of the sternum: cranial, at the 1^st^-5^th^ sternebrae (atypical PE); and caudal including the caudal sternum between the 5^th^-8^th^ sternebrae (typical PE) (Fig 1) [23, 26]. For the purpose of this study, the length of the thoracic depression was defined as short if the inward deviation of the sternum included three sternebrae. If the inward deviation of the sternum included four or more sternebrae, the length of the thoracic depression was defined as long (Fig 1).

**Fig 2:**
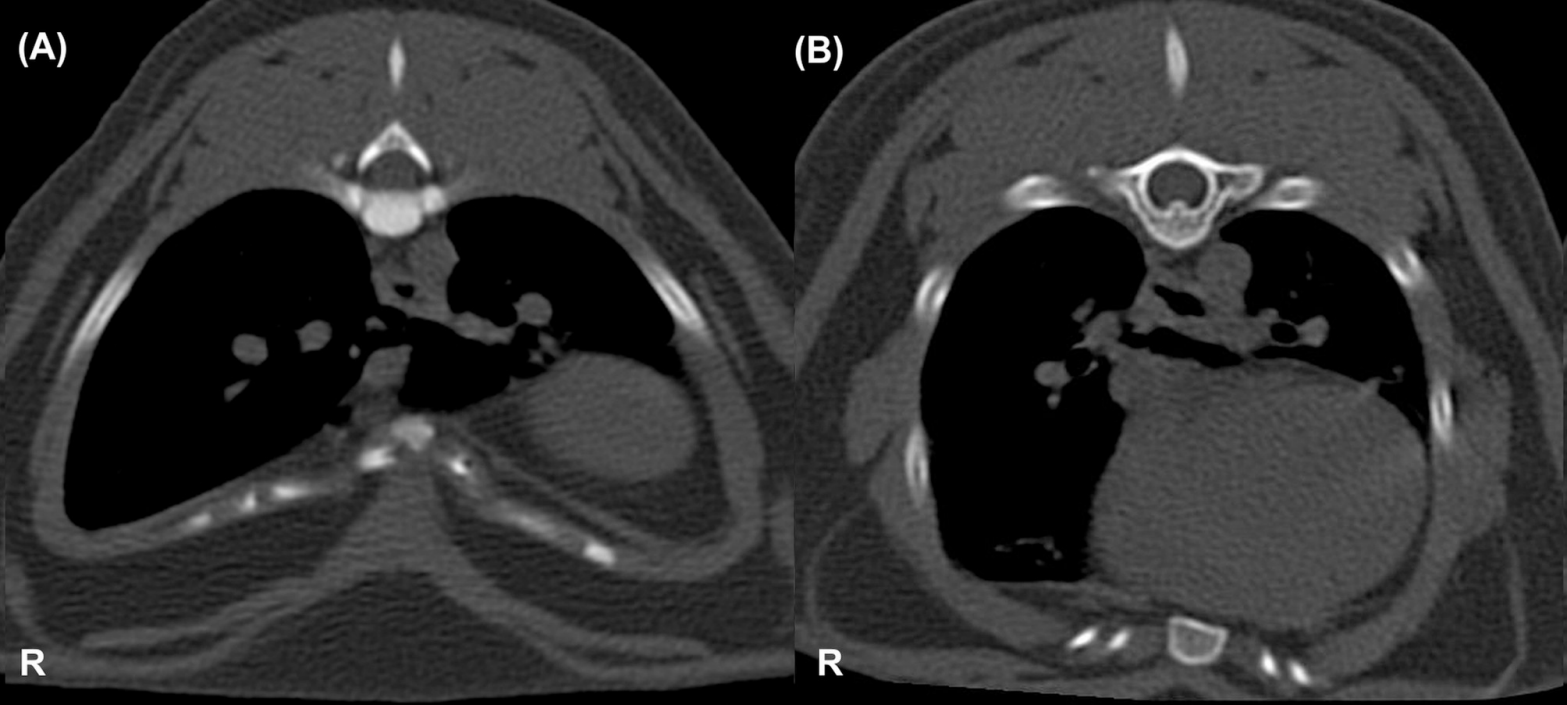
Transverse CT images, the level of the 10^th^ (image A) and 8^th^ thoracic vertebra (image B) of two different cats. Image (A) shows symmetric PE. Image (B) demonstrates asymmetric PE, with asymmetry to the right (the thoracic depression is placed slightly lateral to the midline, and the right hemithorax is smaller than the left hemithorax).

**Fig 3:**
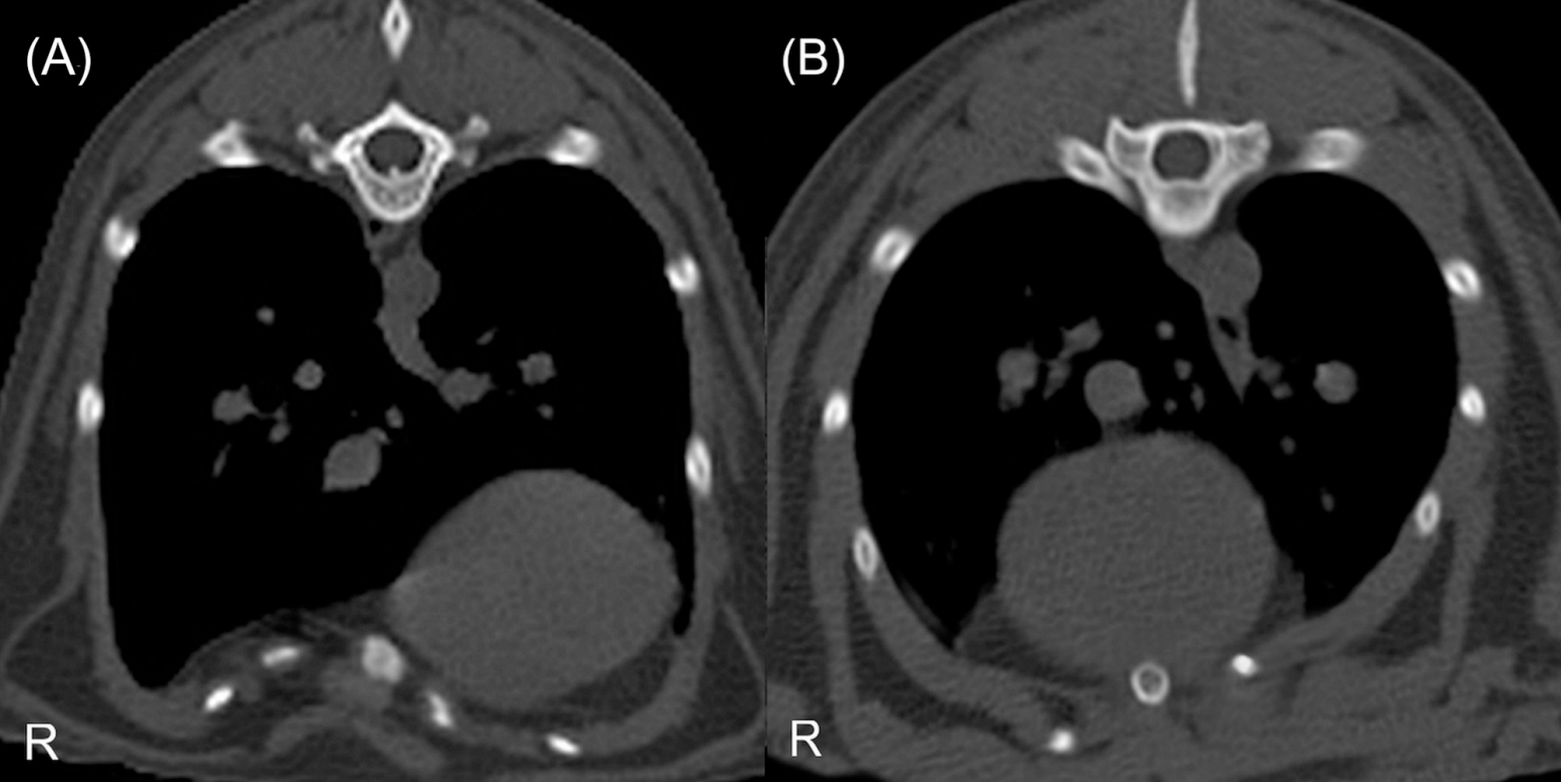
Transverse CT images, the level of the 9^th^ (image A) and 10^th^ thoracic vertebra (image B) of two different cats with asymmetric PE. Image (A) shows 2A PE (the sternum is located in the midline, but the centre of the thoracic depression is located in the right hemithorax). Image (B) presents 2B PE (the centre of the depression is located in the midline, but the left side of the wall of the depression is more severely depressed than the right).

Based on transverse CT images of the thorax, it was determined whether the deepest point of the thoracic depression was located in the midline or was lateralised (Fig 2) [6, 24, 27]. Symmetric PE (PE 1) was defined as thoracic depression located in the midline. Asymmetric PE (PE 2) was defined as thoracic depression located lateral to the midline. Asymmetric PE has been divided into subgroups (S1 Appendix, Fig 3) [27].

**Fig 4:**
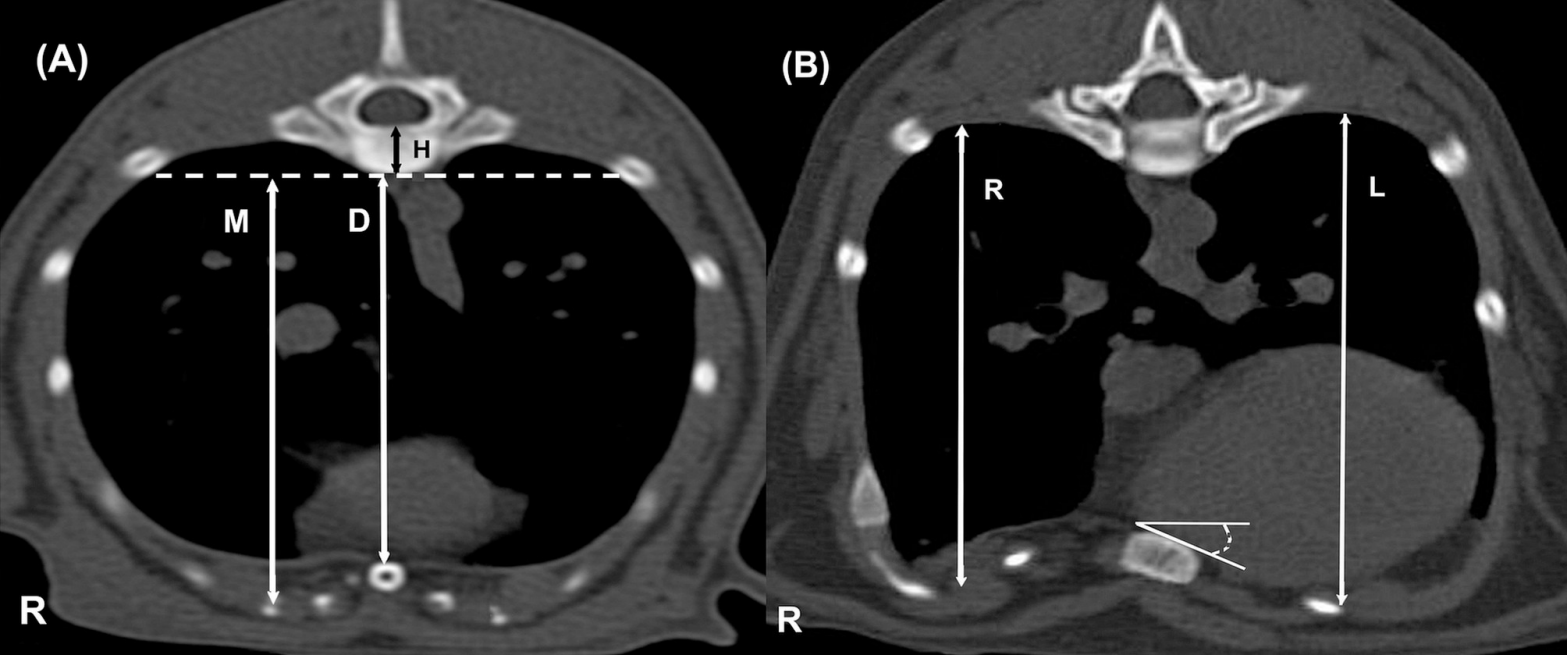
Demonstration of measurements made using a digitizer on transverse plane CT images of the chests of two different cats. D–the shortest distance between the vertebra and the sternum, H–the dorsoventral diameter of the body of the same vertebra, M–the maximum distance between the ventral spine and the inner margin of the most ventral portion of the rib cage, R–the maximum dorsoventral diameter of the right hemithorax, L–the maximum dorsoventral diameter of the left hemithorax; the angle between the sternum and the horizontal line indicates the sternal torsion angle.

According to the principles applicable in veterinary and human medicine [24, 26, 28–30], the VI, CI, AI and the degree of sternal torsion was calculated at the most depressed point of the thorax wall. For this purpose, the following were measured in the transverse plane CT images (Fig 4):

1. The shortest distance between the vertebra and the sternum = D.
2. The dorsoventral diameter of the body of the same vertebra = H.
3. The maximum distance between the ventral spine and the inner margin of the most ventral portion of the rib cage = M.
4. The maximum dorsoventral diameter of the right hemithorax = R.
5. The maximum dorsoventral diameter of the left hemithorax = L.
6. The degree of sternal torsion (calculated considering the angle between the sternum and the horizontal line).

On the basis of the abovementioned measurements, the following were calculated:

VI=(D+H)÷H Eq 1

CI=(M−D)÷M×100% Eq 2

AI=R÷L×100 Eq 3

The severity of the PE was determined from the VI value. A VI value of less than 6 meant severe PE, a value between 6 and 8.99 meant moderate PE and a value between 9 and 12.59 meant mild PE [5, 10, 14].

An AI value of less than 100 meant asymmetry to the right of the thoracic cavity, whereas an AI value of greater than 100 meant asymmetry to the left of the thoracic cavity [21, 24].

### Statistical analysis

The ANOVA analysis of variance (together with Tukey’s post-hoc HSD test) was used to examine differences in CI values with respect to grade VI. A value of p < 0.05 was assumed to indicate statistically significant differences or relationships. The statistical studies were carried out, using Statistica 9.1 computer software (StatSoft, Poland).

## Results

CT scans of 14 cats (nine males and five females) met the required criteria and were included in the study; 10 cats (71%) were domestic short hair cats. PE was also diagnosed in three (21.4%) Maine Coons and 1 (7.1%) Devon Rex. The mean age of the animals examined was 6.9 years (range from 10 months to 16 years, standard deviation of 4.85 years). The CT findings are summarised in Table 1.

**Table 1:** Imaging findings on thoracic CT in 14 cats.

Disorders			Cats
PE	Location/Type	Cranial/atypical	7 (50%)
		Caudal/typical	7 (50%)
	Length	long	5 (35.7%)
		short	9 (64.3%)
	PE symmetry	1	8 (57.1%)
		2	6 (42.9%)
Thoracic asymmetry		R	4 (28.6%)
		L	2 (14.3%)
Sternal torsion			6 (42.9%)

PE–pectus excavatum, 1 –symmetric PE, 2 –asymmetric PE, R–asymmetry to the right of the thoracic cavity, L–asymmetry to the left of the thoracic cavity.

Seven cats (50%) were diagnosed with atypical (cranial) PE while the remaining cats (n = 7, 50%) had typical (caudal) PE. Short PE (n = 6, 64.9%) was almost twice as frequent as long PE. Asymmetric PE was reported in six cats (42.9%). Eleven cats (78.6%) had severe or moderate PE. The CI in the study group ranged from 12.20 to 32.11. The AI value was between 90 and 107, i.e. none of the cats exceeded 10% asymmetry. Asymmetry to the right of the thoracic cavity (n = 4, 66.7%) was reported twice as often as asymmetry to the left of the thoracic cavity. Sternal torsion was found in six cats (42.9%), which was not greater than 30° in any of the cats.

Statistically significant differences were shown in the CI values in cats affected by mild, moderate and severe PE (p = 0.02). Detailed analysis showed statistically significant differences in CI values between the group of cats with mild PE and the group of cats with severe PE (p = 0.023) and between the group of cats with moderate PE and the group of cats with severe PE (p = 0.038). In both cases, higher CI measurements were noted in the group of cats with severe PE (Table 2). No statistically significant difference was found in the CI values between the mild PE and moderate PE groups (p = 0.618).

**Table 2:** Comparison of correction index (CI) values in cats affected by mild (n = 3), moderate (n = 8) and severe (n = 3) PE.

Parameter	mild PE		moderate PE		severe PE	
	Mean (Min-Max)	Median (Q1- Q3)	Mean (Min-Max)	Median (Q1-Q3)	Mean (Min-Max)	Median (Q1-Q3)
CI	12.70 (12.43–12.94)	12.74 (12.43–12.94)	15.36 (12.20–19.72)	14.42 (13.29–17.78)	23.32 (16.04–32.11)	21.81 (16.04–32.11)

PE–pectus excavatum, CI–correction index, Q1 –lower quartile, Q3 –upper quartile.

## Discussion

This is the first study that focuses on such a large variety of CT features of PE in cats. So far, in animals the PE location and the degree of PE have usually been assessed on the basis of radiographic tests. According to us other features are also noteworthy. The length and symmetry of the defect, thoracic symmetry/asymmetry, and possible presence of sternal torsion may prove important when comparing the results of studies in animals affected by PE. Their evaluation, except the length of PE, is only possible on the basis of CT images.

The high percentage of atypical location of PE in the group of cats studied (50%) deserves special attention. In recent years, the first studies indicating dorsal deviation in the cranial-sternal region have appeared in veterinary literature [23, 26]. Based on chest radiography it was reported an atypical PE location in 42% of affected brachycephalic dogs [23]. Typical and atypical PE locations were equally frequent in the group of cats studied. This is an important observation, as it was demonstrated that the severity of clinical symptoms in dogs with PE depends mainly on the site/type of deviation rather than the degree of deviation.

The present study indicated a more frequent occurrence of short PE. However, in 35.7% of the cats examined, defect depression included more than three sternebrae. Single case descriptions of long-term sternal depression in cats with moderate PE, usually in combination with surgical treatment, are available in veterinary literature [6, 8]. However, unlike in human medicine [24, 31–33], in veterinary medicine, this feature is often not taken into consideration. Perhaps this is the reason why the cases of the long thoracic depression that have been revealed in cats were not related to severe PE. In humans, the extremely long severe PE which involves the upper chest may impact the function of the esophagus [24].

Asymmetric PE was reported in 42.9% of the cats examined. Such a large percentage of defect asymmetry draws attention to the need for evaluating chest wall malformations on CT scans. Unlike X-rays, CT scans allow the evaluation of the defect in different planes. Until now, asymmetric PE has been described in a cat in only one study, in which the asymmetry of the defect has been described in 50% of cats with PE [6]. The thoracic tomographic analysis in cats with PE also demonstrated that midline deformities of the xiphoid are associated with more severe clinical symptoms. There are certain differences between this study and the present study. These relate primarily to the age of the animals studied and the positioning of the animals for examination. However, it seems that these differences should not affect the localisation and symmetry of PE. A completely new observation is that in the group of cats with asymmetric PE, different morphological types of defects can be distinguished, similar to those described in people by Park et al. [27].

In the present study most animals had moderate or severe PE (11 cats). Similarly, in other studies the most commonly described cases of cats with PE are those related to moderate to severe PE [6, 7, 9–11, 14, 17]. The remaining 21.5% of cats, with mild PE, are particularly interesting, as this is a group of animals affected by PE but not taken into account in previous studies. Its inclusion may prove important in research into the aetiology of the disease.

In people with PE, an objective assessment of thoracic malformations through CT scanning can be performed using a number of indices, including: the pectus index, the AI, the flatness index, the sternal torsion angle (discussed below), and the angle of Louis [31]. When calculating the pectus index and the flatness index, the width of the chest is taken into account, which may lead to an incorrect assessment, as in the case of FSI [19–22]. The adaptation of the angle of measurement between the manubrium and the body of the sternum to veterinary needs, however, is made difficult on account of the differences in the build of the sternum in people and cats. We propose to use the coefficients used successfully in human medicine to accurately assess PE: CI and AI. They are easy to measure and recreate in repeated measurements. The CI corresponds to the percentage of chest depth that should be corrected [22, 29, 30]. While the VI also determines the degree of reduction of the thoracic depth [2, 4, 23], the CI value remains independent of the dorsoventral diameter of the vertebral body used to calculate the VI, the dimension of which may depend on, for example, the breed of the tested animal. The CI also remains independent of the width of the thorax used to calculate the FSI, as mentioned in the introduction. The minimum thoracic height index and dorsal sternum depression, used in PE measurements in cats, seems to be useful in the assessment of efficacy of the surgical adjustment performed in particular patients [8, 11]. In humans, it is believed that a CI value greater than 10% confirms the presence of PE [21, 22]. In the current study involving cats affected by PE, the CI value was greater than 12.2%.

The AI represents the difference between the depth of the right hemithorax and the depth of the left hemithorax [21, 24]. Neither the VI nor CI accounted for the aspect of thoracic asymmetry. In humans the omission of the AI value during treatment planning may change the outcome of the corrective surgery [21, 24]. In this study, the size of thoracic asymmetry did not exceed 10%. It is believed that in humans, a higher degree of thoracic asymmetry affects the positioning and compression of internal organs in the chest and abdominal cavity and exacerbates clinical symptoms [24].

In the current study, asymmetry to the right of the thoracic cavity was observed more frequently. Similar results were obtained in the CT scans of PE in kittens [6]. This observation is consistent with our expectations. It is believed that in young people with PE, the negative pressure in the thoracic cavity created during inhalation is conducive to deeper movement of the sternum to the interior of the chest. The heart, located in the left hemithorax, prevents further depression and therefore dextral sternal deviation entails [34]. In this study, asymmetry in the thoracic cavity was always combined with asymmetric PE. However, other factors that may cause such an asymmetry, such as scoliosis, should always be taken into account in the overall assessment of thoracic cavity asymmetry [24, 31]. Only animals without thoracic spine deformities were included in the study group.

CT scans revealed sternal torsion in 42.9% of cats with PE. This feature may prove to be particularly important when planning the operation. Experience in human medicine shows that in the case of strong rotation (more than 30°), sternal deformation may remain visible after surgery [24]. Moreover, there has been a report indicating the possibility of sternal torsion increasing during growth [34].

The study confirmed statistically significant differences in the CI values between the groups of cats with different degrees of PE severity. High CI values in cats with severe PE allowed us to distinguish this group from animals with mild PE and moderate PE. Further studies are needed to demonstrate the differences in the CI values between the mild PE and moderate PE groups.

The retrospective nature of this study imposes limitations, mainly due to the relatively occasional incidence of PE in cats. This results in a small number of included in the study. Further multi-centre trials may be able to address this problem. The next step in the study would be the correlation of individual PE types and estimated indicators with the occurrence and severity of clinical symptoms in cats.

## Conclusion

One of the main benefits of CT examination is the ability to reveal different types of PE. CT examination shows location, length and symmetry of PE and the possible presence of thoracic asymmetry or sternal torsion. These features of PE should be considered as they may influence the incidence and severity of clinical symptoms in cats. The criteria that quantify thoracic wall deformities suggested by the authors (CI and AI) allow a better overall comparability of cats with PE. Taking into account several variants of PE in genetic tests would enable us to understand the aetiology of PE more thoroughly.

## Supporting information

S1 Appendix Raw data from thorax tomography. DSHC–domestic short hair cat, MC–Main Coon, DR–Devon Rex, PE–pectus excavatum, 1 –symmetric PE, 2 –asymmetric PE, 2A –sternum is in the midline, but the centre of the thoracic depression is in the parasternal sections of the ribs off to one side, 2B –the centre of the depression is located in the centre of the sternum, but one side of the thorax wall is more depressed than the other, 2C –combination of both type 2A PE and 2B PE, R–asymmetry to the right of the thoracic cavity, L–asymmetry to the left of the thoracic cavity, VI–vertebral index, CI–correction index, AI–asymmetry index.
(XLSX)
